# Treatment for Insect Bites

**Published:** 1897-12

**Authors:** 


### TREATMENT FOR INSECT BITES.

Children, without doubt, suffer more severely from the effect of the bites of insects than adults, and it is comforting at all times to read of any description of treatment that may give relief. Still the truth must be confessed that many of the advertised remedies do not act up to expectation; their curative or soothing qualities are grossly exaggerated, so that when one hears on really reliable authority of an efficient anti-inflammatory, one feels tempted, even in the face of former unlucky experiences, to give it a trial. Such a panacea for insect bites is said to be found in ichthyol. W. Ottinger says of ichthyol, in *Munchener Med. Wochenschrift*, 1896: "In the case of bites of flies, bees, wasps, etc., the application of ichthyol quickly causes the inflammatory phenomena to abate, and in a few minutes all feeling of pain, burning and itching ceases. It is best applied pure, a thick layer being laid on with a brush." During this present summer the mosquito plague, as well as that of other insects, has been, owing to the excess of moisture, exceptionally troublesome, while to children in parts of the country it has been most dangerous.—*Pediatrics*.

